# Forskolin Stimulates Estrogen Receptor (ER) *α* Transcriptional Activity and Protects ER from Degradation by Distinct Mechanisms

**DOI:** 10.1155/2022/7690166

**Published:** 2022-05-09

**Authors:** Houng-Wei Tsai, Vicky Y. Lin, Margaret A. Shupnik

**Affiliations:** ^1^Department of Biological Sciences, California State University, Long Beach, CA 90840, USA; ^2^Division of Endocrinology and Metabolism, Department of Internal Medicine, University of Virginia School of Medicine, Charlottesville, VA 22908, USA

## Abstract

Estradiol action is mediated by estrogen receptors (ERs), *a* and *ß*. Estradiol binding initiates ER-mediated transcription and ER degradation, the latter of which occurs via the ubiquitin-proteasome pathway. Inhibition of proteasome activity prevents estradiol-induced ER*α* degradation and transactivation. In ER-positive GH3 cells (a rat pituitary prolactinoma cell line), forskolin, acting via protein kinase A (PKA), stimulates ER*α* transcriptional activity without causing degradation, and proteasome inhibition does not block forskolin-stimulated transcription. Forskolin also protects liganded ER*α* from degradation. In the current study, we first examined ER*α* and ER*β* transcriptional activity in ER-negative HT22 cells and found that forskolin stimulated ER*α*-, but not ER*β*-dependent transcription, through the ligand-binding domain (LBD). We also identified four mutations (L396R, D431Y, Y542F, and K534E/M548V) on the ER*α* LBD that selectively obliterated the response to forskolin. In GH3 cells, transfected ER*α* mutants and ER*β* were protected from degradation by forskolin. Ubiquitination of ER*α* and ER*β* was increased by forskolin or estradiol. ER*α* ubiquitination was diminished by a mutated ubiquitin (K48R) that prevents elongation of polyubiquitin chains for targeting the proteasome. Increased ER*α* ubiquitination was not affected by the deletion of the A/B domain but significantly diminished in the F domain deletion mutant. Our results indicate distinct and novel mechanisms for forskolin stimulation of ER*α* transcriptional activity and protection from ligand-induced degradation. It also suggests a unique mechanism by which forskolin increases unliganded and liganded ER*α* and ER*β* ubiquitination but uncouples them from proteasome-mediated degradation regardless of their transcriptional responses to forskolin.

## 1. Introduction

Estrogens exert their actions through estrogen receptors (ERs), ER*α* and ER*β*, to regulate a variety of physiological functions of the cardiovascular, endocrine, musculoskeletal, nervous, and reproductive systems [1, 2]. ERs are members of the steroid/nuclear receptor superfamily with four major functional domains, including the amino-terminal, ligand-independent transactivation domain (activation function-1, AF-1), the central DNA-binding domain (DBD), the hinge region, and the carboxyl-terminal ligand-binding domain (LBD)/ligand-dependent transactivation (activation function-2, AF-2) [1, 2]. In the classical pathway, ERs undergo a conformational change once bound by estrogens (activation), forming a dimer and then binding to estrogen response elements (EREs) in the transcriptional regulatory regions of target genes [1–3]. Acting as bridging proteins, liganded ERs dynamically recruit transcriptional coactivators and components of the RNA polymerase II transcription initiation complex to enhance target gene transcription [3–5]. Concomitant with increased ER*α* transcriptional activity, ligand binding also causes ER*α* protein degradation [6–10]. Deletion of the LBD of human ER*α* or mutations within this domain that prevent ligand binding (G521R) and/or the activation of helix 12 for coactivator interaction (D538A, L539A/L540A, D538A/E542A/D545A) decrease ligand-induced proteolysis [6, 9, 11, 12]. These data suggest that specific conformational changes in liganded ER*α* LBD are important not only for transcriptional activity but also for receptor degradation.

Estradiol-induced ER*α* protein degradation is mediated by the ubiquitin-proteasome pathway [6, 8–10]. In this pathway, the target proteins are first covalently conjugated with ubiquitin on the lysine residues by three classes of enzymes, including ubiquitin-activating enzymes (Uba), ubiquitin-conjugating enzymes (Ubc), and ubiquitin ligases [13, 14]. Free ubiquitin is then added to the lysine 48 (K48) on the ubiquitin conjugated to target proteins, and this process is repeated to form a polyubiquitination chain on the substrate protein, which is implicated in targeting the proteins to the proteasome for degradation [15–18]. Several studies have shown that ER*α* becomes ubiquitinated in the presence of estradiol or selective ER modulators (SERMs) [9, 19].

Multiple lines of evidence indicate a functional linkage between ligand-dependent ER transcription and the ubiquitin-proteasome system. Prevention of ER*α* degradation by proteasome inhibitors, such as MG132 and lactacystin, disrupts estradiol-induced ER*α* transactivation in HeLa cervical cancer cells, MCF7 breast cancer cells, and GH3 pituitary cells [6, 8, 20]. A temperature-sensitive mutation of the Uba, disrupting protein ubiquitination, abolishes ligand-induced ER*α* degradation and ER*α*-mediated transcription [6]. Chromatin immunoprecipitation of estradiol-sensitive gene promoters demonstrates that cyclical occupancy of EREs by liganded ER*α* is regulated by the proteasome and required for the transcriptional responsiveness of ER*α* to estradiol [20–22]. Additionally, the ubiquitin ligase E6-associated protein (E6-AP) and suppressor of gal 1 (SUG1/TRIP1), an ATPase subunit of the 26S proteasome complex, are both reported to act as transcription cofactors of ERs [23–25]. Thus, the ligand-dependent transactivation of ER*α* is tightly linked to its degradation through the ubiquitin-proteasome pathway, but it is unclear if similar changes in conformation and the activation-degradation linkage also hold true for ER*β*.

Ligand-independent activation of ER has been described in several cell types, including the uterus, cervix, and pituitary, and there are clearly both context- and signaling-pathway-dependent contributions [26–28]. For example, mitogen-activated protein kinase (MAPK) stimulation of ER*α* activity occurs through the AF-1 region and potentially via direct phosphorylation of the receptor and coactivators [27, 29–31], whereas either GnRH acting via protein kinase C (PKC)/MAPK pathways [32] or cAMP acting via protein kinase A (PKA) stimulated transcriptional activity of pituitary ER*α* [7, 8]. Much less is known about the mechanisms by which these ligand-independent pathways function in ER activation and turnover, and it is unclear whether direct posttranslational modification and/or conformational changes of ER are involved.

We have previously shown that forskolin, which activates adenylyl cyclase and increases intracellular levels of cAMP, stimulates ER-mediated transcriptional activity in rat lactotroph GH3 cells through PKA without causing ER*α* degradation and that inhibition of proteasome activity had no effect on forskolin-stimulated transcription [8]. The dissociation of forskolin-stimulated, ER-mediated transcription from both receptor degradation and the requirement for proteasome activity suggests important differences between mechanisms of transactivation by forskolin and estradiol. Forskolin also protects liganded ER*α* from degradation, suggesting that PKA-dependent pathways may generally stabilize ER*α*. In this study, we examined the hypothesis that forskolin-induced rat pituitary ER*α* activation and protection of rat ER*α* from degradation occurred through separate mechanisms by dissecting the molecular events involved in receptor activation and degradation. We found that only ER*α*, not ER*β*, was stimulated by forskolin, and several mutations in the LBD selectively impacted ER*α* transcriptional activation by forskolin. However, forskolin treatment protected both ER*β* and the transcriptionally inactive mutated ER*α* from degradation after ligand binding, demonstrating that these two molecular processes can be uncoupled.

## 2. Materials and Methods

### 2.1. Chemicals and Antibodies

Cycloheximide and 17*β*-estradiol were purchased from Sigma (St. Louis, MO), and forskolin was purchased from Tocris (Ellisville, MO). Tumor necrosis factor *α* (TNF*α*) and ubiquitin carboxyl-terminal hydrolase L1 (UCH-L1) inhibitor as well as lactacystin and MG132 were purchased from Calbiochem (San Diego, CA) and BIOMOL Research Laboratories Inc. (Plymouth Meeting, PA), respectively. Hemagglutinin (HA) and His6 antibodies were purchased from the Hybridoma Core, University of Virginia (Charlottesville, VA) and Novagen (Madison, WI), respectively. The antibody against amino acids 586–600 of the rat ER*α* (C1355), generated by our lab, was characterized previously [33].

### 2.2. Plasmids

Rat ER*α* and ER*β* cDNAs were subcloned into the pcDNA3.1 expression vector (Invitrogen, Carlsbad, CA) as described previously [34, 35]. To generate HA-tagged ER, ER*α* and ER*β* cDNAs were excised from the pcDNA3.1 vector with BamHI and EcoRI and then ligated into the pKH3 expression vector, which contained three copies of the HA tag immediately 5′ to the multiple cloning site (graciously provided by Dr. Ian Macara, Vanderbilt University, Nashville, TN) [36]. The pcDNA3.1 and pKH3 vectors both carried the transgene under the control of a cytomegalovirus (CMV) promoter. Site-directed mutagenesis was used to introduce specific amino acid substitution mutations to ER*α* DBD and LBD using the QuickChange Site-directed Mutagenesis Kit (Stratagene, La Jolla, CA). A list of the mutations is selected because the same mutations on the equivalent residues of human ER*α* have been reported to (1) interfere PKA phosphorylation (S241E and S523A) [37, 38], (2) alter transcriptional activity in response to estradiol or SERMS (D356V, K367A, L377R, V381R, E385Q, D431Y, Y531A, K534E, Y542E, Y542F, and Y542S) [39–41], or (3) perturb LBD helical structures for ligand binding and/or cofactor interaction (N353A, L396R, L408A, N460A, D478A, H493A, and M548V) (in consultation with Dr. Fraydoon Rastinejad). Besides, the double mutant of K534E/M548V was created unexpectedly when cloning rat ER*α* cDNA to an expression vector, and these substitution mutations were confirmed by DNA sequencing.

In addition, we also used a series of domain deletion mutants derived from the HA-ER*α* construct to examine the contribution of the individual regions towards ER transcriptional activity and stability. To generate HA-ER*α* deletion mutants lacking the E/F (ΔE/F), helix 12 and F (ΔH12/F), or F (ΔF) domains, a TGA stop codon was introduced immediately before each of those domains. The HA-ER*α* A/B deletion (ΔA/B) was constructed by introducing a BamHI restriction enzyme site at the end of the B region of ER*α* cDNA, followed by excising and subcloning the modified cDNA into the pKH3 vector with BamHI and EcoRI. The HA-tagged ubiquitin vector, containing eight copies of ubiquitin, was kindly provided by Dr. Deborah Lannigan (Vanderbilt University) [42, 43]. His6- and HA-tagged ubiquitin vectors, including both wild-type and K48R, were provided by Drs. Ron Kopito (Stanford University, Sanford CA) and Ze'ev Ronai (Burnham Institute for Medical Research, La Jolla, CA), respectively [44, 45]. The pGL3-2ERE reporter containing two ERE consensus sequences followed by a prolactin TATA box immediately upstream of the firefly luciferase gene was used to analyze the transcriptional activity of ER [46].

### 2.3. Luciferase Reporter Assays for Measuring ER-Mediated Transcription

Mouse hippocampal HT22 cells, lacking endogenous ER*α* or ER*β*, were maintained in Cellgro® Dulbecco's modified Eagle's medium (DMEM; Mediatech/Fisher, Herndon, VA) containing 10% fetal bovine serum (Gibco/Invitrogen, Grand Island, NY) and 100 U/ml penicillin and 100 *µ*g/ml streptomycin (Gibco/Invitrogen). Cells were plated in phenol red-free DMEM with 5% charcoal-stripped newborn calf serum (sNCS) at the density of 1 × 10^5^ cells per ml in 18 mm well plates. Cells were transiently transfected with the pGL3-2ERE reporter (500 ng/well) plus a control or ER expression vector (10 ng/well) for 18–22 h with FuGENE 6 (Roche, Indianapolis, IN) [8]. Cells were then treated with vehicle, 10 nM estradiol, or 1 *μ*M forskolin for 24 h, then washed with phosphate-buffered saline, and then collected in 200 *μ*l of 1 × Cell Culture Lysis Reagent (Promega, Madison, WI) and later assayed for luciferase activity using a Turner TD-20e luminometer (Sunnyvale, CA). Total protein levels of individual lysates were also determined by Bio-Rad Protein Assay (Bio-Rad Laboratories, Richmond, CA). Luciferase activity from each sample was normalized to total lysate protein levels as described [8]. Each treatment was performed in triplicate, and experiments were repeated at least three times.

### 2.4. Immunoblotting for Measuring Levels of Endogenous and Transfected ERs

GH3 cells, a rat pituitary-derived cell line, were plated in DMEM with 5% sNCS at 1.2 × 10^6^ cells per 35 mm well. In some studies, cells were transfected with control or HA-tagged ER expression vectors (400 ng/well) with Lipofectamine™ 2000 (Invitrogen) for 18–20 h. Cells were pretreated with vehicle or cycloheximide (20 *μ*g/ml) for 30 min, followed by vehicle, 10 nM estradiol, 1 *μ*M forskolin, or both for 6h. Cells were then collected in gel loading buffer as previously described [8, 46]. Total protein levels of the lysates were determined using BCA Protein Assay (Pierce Chemical Co., Rockford, IL). Individual lysates (∼30 *μ*g each) were separated on 8% polyacrylamide-SDS gels and transferred to nitrocellulose membranes (Bio-Rad). Endogenous ER*α* was detected with ER*α* antibody, C1355 (1 : 7,500), while transfected HA-tagged ER was detected with HA antibody at a concentration of 1 : 10,000. After rinsing, blots were then incubated in a horseradish peroxidase (HRP)-conjugated donkey anti-rabbit IgG (1 : 10,000; Amersham Pharmacia Biotech, Arlington Heights, IL) or goat anti-mouse IgG secondary antibody (1 : 10,000; Jackson ImmunoResearch Laboratories, West Grove, PA) for 1 h, followed by incubation in SuperSignal® West Pico Chemiluminescence (Pierce Chemical Co.) and detection on Kodak X-OMAT X-ray film (Kodak Co., Rochester, NY). The same blots were reprobed with the *β*-actin antibody at 1 : 50,000 (Sigma), then incubated in an HRP-conjugated goat anti-mouse IgG secondary antibody (1 : 50,000; Jackson ImmunoResearch Laboratories) for 1 h and chemiluminescent detection. With a densitometer, the intensities of ER and *β*-actin bands on each film were measured and analyzed using ImageQuant (Molecular Dynamics, Inc., Sunnyvale, CA). Relative ER protein level of each sample was calculated by normalizing the intensity of ER to that of *β*-actin and expressed as a percentage of vehicle-treated controls (as 100%). Each experiment was performed in duplicate wells and repeated at least three times.

### 2.5. Immunoprecipitation and Immunoblotting for Detecting ER Ubiquitination

GH3 cells plated at a density of 8 × 106 cells per 100-mm Corning® cell culture dish (Fisher) were transfected with the expression vectors for HA- or His6-tagged ubiquitin (4 *μ*g/dish) for 18–20 h. Cells were pretreated with vehicle or MG132 (40 *μ*M) for 1 h, followed by treatment of vehicle, estradiol, forskolin, or both for 6 h. Cells were then collected in M-PER® Mammalian Protein Extraction Reagent (Pierce Chemical Co.) containing N-ethylmaleimide (Sigma) and a cocktail of protease inhibitors. Total lysate protein levels were similarly determined with BCA Protein Assay (Pierce Chemical Co.). Lysates of 500 *μ*l (1 *μ*g/*μ*l) were incubated with the antibodies for ER*α* (1 : 250) at 4°C for 18 h, or the antibodies against HA or His6 tags (1 : 100) at 4°C for 18 h followed by Protein *G* PLUS-Agarose (40 *μ*l; Santa Cruz Biotechnology Inc., Santa Cruz, CA) at 4°C for 1 h. Lysates were centrifuged, and the pallets were washed twice with RIPA buffer, followed by separated on 8% polyacrylamide-SDS gels, transferred to nitrocellulose membranes, and subjected to immunoblotting for HA or His6.

### 2.6. Statistical Analyses

The data of luciferase activities and ER protein levels were statistically analyzed by one-way or two-way analysis of variance (ANOVA) to reveal the effects of treatments and ER mutations. A confidence level of *P* < 0.05 was considered significant. If there was a significant main effect or interaction, Tukey's wholly significant difference (WSD) post hoc test was further used for multiple pairwise comparisons.

## 3. Results

### 3.1. Forskolin Increases Transcriptional Activity of ER*α*, but Not ER*β*, Which Is Independent of Proteasome Activity

**Figure 1:**
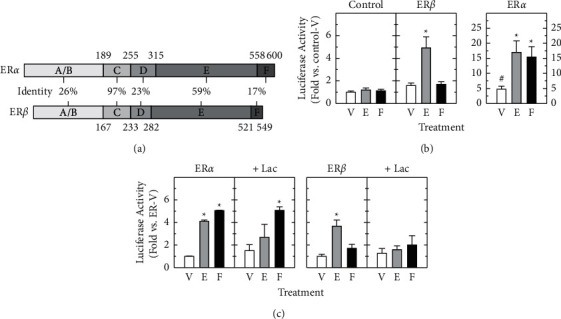
Forskolin increases ER*α*-, but not ER*β*-mediated transcription, and forskolin stimulation of ER*α* transactivation does not require the proteasome pathway. (a) The panel shows the domain organization and sequence homology of rat ER*α* and ER*β*. (b) HT22 cells were transiently transfected with pGL3-2ERE luciferase reporter (500 ng) plus a control (pcDNA3.1), rat ER*α*, or ER*β* expression vector (10 ng). These cells received the treatment of either vehicle (V), estradiol (E, 10 nM), or forskolin (F, 1 *μ*M) for 24 h. Luciferase activity (mean ± SEM) was normalized and expressed as fold stimulation over the vehicle-treated controls (as 1 fold) from at least three independent experiments with triplicate samples. ^*∗*^, *P* < 0.05 vs. vehicle-treated groups (V) with the same ER expression vector. ^#^, *P* < 0.05 vs. vehicle-treated group with the empty expression vector (Control-V). (c) HT22 cells, transfected with pGL3-2ERE luciferase reporter plasmid (500 ng) plus a rat ER*α* or ER*β* expression vector (10 ng), were pretreated with either vehicle or lactacystin (+Lac, 1 *μ*M), followed by vehicle (V), estradiol (E), or forskolin (F). Normalized luciferase data were expressed as the fold stimulation over vehicle-treated cells carrying the same ER construct (without lactacystin) (1 fold) and expressed as the mean ± SEM from three independent experiments performed in triplicate. ^*∗*^, *P* < 0.05 vs. vehicle-treated groups (V) with the same ER expression vector (without lactacystin).

Figure 1(a) depicts the protein domains and sequence similarity of rat ER*α* and ER*β*, which are composed of 600 and 549 amino acids. Between the two ER subtypes, the amino-terminal A/B domain shares a 26% amino-acid identity, while the central C region is near-identical (97%), the hinge, or D, domain shows 23% identity, whereas in the carboxyl terminal, the E and F domains share 59% and 17% amino-acid homology, respectively.

We have previously used GH3 cell line as a model to demonstrate ER-mediated transactivation in response to forskolin, and inhibition of 26S proteasome activity does not disrupt the effect of forskolin on ER transcriptional activity [8]. GH3 cells express both ER*α* and ER*β* although the former seems to be the major subtype [8, 47, 48]. Thus, we first investigated the ER subtype specificity of forskolin-stimulated transactivation using ER-negative HT22 cells transiently transfected with a pGL3-2ERE-luciferase reporter plus either a control, rat ER*α*, or rat ER*β* expression vector. There was no stimulation of the ERE-reporter activity by estradiol (1.20 ± 0.18 fold) or forskolin (1.12 ± 0.14 fold) in the absence of ER (Control; Figure 1(b), left panel). Cells transfected with ER*α* showed increased basal transcriptional activity (4.77 ± 1.01 fold) as well as both estradiol- and forskolin-stimulated ER transcriptional activity (17.01 ± 3.82 and 15.40 ± 3.48 folds, respectively; *P* < 0.05) (Figure 1(b), right panel). In ER*β*-transfected cells, estradiol was able to stimulate the ERE-reporter activity (4.92 ± 0.97 fold) (*P* < 0.05), not forskolin (1.7 ± 0.23 fold) (Figure 1(b), middle panel). Our data demonstrate that forskolin stimulation of rat ER transcriptional activity is ER subtype-specific.

Since inhibition of 26S proteasome activity by lactacystin disrupted ligand-dependent, not ligand-independent, transcriptional activity of endogenous ERs in GH3 cells [8], we then examined if estradiol and forskolin stimulation of transfected ER*α* and ER*β* similarly required the 26S-proteasome pathway. HT22 cells, transfected with an ERE-luciferase reporter plus either an ER*α* or ER*β* expression vector, were pretreated with lactacystin (10 *μ*M) for 1h, followed by treatment of estradiol or forskolin for 24 h and then measurement of ERE reporter activity. In agreement with Figure 1(b), estradiol stimulated transcriptional activity of ER*α* (4.10 ± 0.13 fold) and ER*β* (3.67 ± 0.54 fold) (*P* < 0.05), which was suppressed by lactacystin at the concentration that had shown to prevent estradiol-induced ER*α* degradation in GH3 cells (Figure 1(c), gray bars). On the other hand, forskolin increased transcriptional activation of ER*α* (5.06 ± 0.03 fold), not ER*β* (1.71 ± 0.35 fold), and unlike liganded ER*α*, the forskolin-stimulated ER*α* transcriptional activity was not affected by lactacystin (5.07 ± 0.31 fold) (*P* < 0.05) (Figure 1(c), closed bars). Our data demonstrate that estradiol stimulation of ER*α*- and ER*β*-mediated transcription requires the proteasome pathway and that the difference in proteasome participation in transcriptional activity indicates distinct mechanisms underlying the ligand-dependent and ligand-independent activation of ER*α*.

### 3.2. Forskolin Increases Transcriptional Activity of ER*α* through the LBD

**Figure 2:**
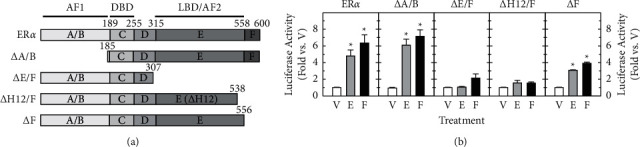
Forskolin increases ER*α*-mediated transcription via the ligand-binding domain (LBD). (a) The upper panel is a schematic representation of full-length rat ER*α* and deletion mutants used in this study. Key functional domains, including the activation function-1(AF-1), the DNA-binding domain (DBD), and the LBD/activation function-2 (AF-2) are indicated on the top. (b) HT22 cells were similarly transfected with pGL3-2ERE luciferase reporter (500 ng) plus an expressing vector carrying wild-type or mutated ER*α* with deletion of A/B (ΔA/B), E/F (ΔE/F), helix12/F (ΔH12/F), and F (ΔF) (10 ng) and treated either vehicle (V), estradiol (E, 10 nM), or forskolin (F, 1 *μ*M) for 24 h. The luciferase activity (mean ± SEM) was normalized and expressed as fold stimulation over vehicle-treated groups carrying the same ER constructs (as 1 fold). ^*∗*^, *P* < 0.05 vs. vehicle-treated groups with the same expression vector.

After showing that the forskolin stimulation of ER transactivation is ER*α*-specific, we then identified which ER*α* region mediated forskolin stimulation. HT22 cells were similarly transfected with an ERE reporter plus an amino-terminal HA-tagged ER*α* expression vector, including full-length and deletion mutants (Figure 2(a)). One amino- and three carboxyl-terminal deletion mutants were used: ΔA/B, which consisted of the C, D, E, and F domains; ΔE/F, which consisted of the A/B, C, and D domains; ΔH12/F, which lacked helix 12 of the LBD and the F domain; ΔF, in which the F domain was deleted. ΔA/B has ligand-binding activity and retains AF-2 transactivation function whereas ΔE/F retains AF-1.

Lack of the A/B region (ΔA/B panel) did not affect estradiol (6.15 ± 0.66 fold) and forskolin (7.21 ± 0.72 fold) stimulation of ER*α* transactivation (*P* < 0.05), whereas deletion of the E/F (ΔE/F panel) regions completely eliminated both estradiol- and forskolin-dependent ER*α* transcriptional activity (1.07 ± 0.07 and 2.14 ± 0.5 folds, respectively) (Figure 2(b)). Deletion of the F region (lacking amino acids 557–600; ΔF panel) reduced but did not abolish ER*α* transactivation in response to estradiol (3.07 ± 0.66 fold) or forskolin (3.92 ± 0.13 fold) while deletion of both the F region and helix 12 (lacking amino acids 539–600; ΔH12/F panel) completely eliminated the responses to estradiol (1.57 ± 0.03 fold) or forskolin (1.55 ± 0.12 fold) (Figure 2(b)). These data indicate that forskolin-stimulated ER*α* transcriptional activity requires the LBD, including the helix 12, and the AF-2 seems to be important for forskolin action on transcriptional activity of unliganded ER*α*.

### 3.3. Mutation of Specific ER*α* LBD Residues Selectively Disrupts Forskolin-Stimulated Transactivation

**Figure 3:**
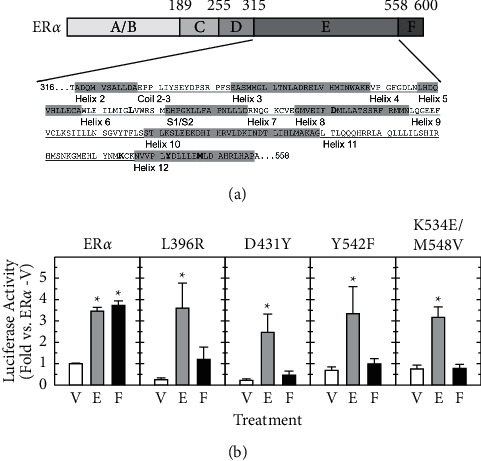
Forskolin-stimulated ER*α* transcriptional activity is mediated by specific residues located within the ligand-binding domain. (a) The upper panel shows rat ER*α* domain structure and the amino-acid sequence of the E domain (amino acids 316–558) with the indication of the alpha helixes (underlined or highlighted) and specific residues (bolded) required for forskolin-stimulated ER*α* transactivation. The helices correspond to those of the human ER*α*, and S1/S2 is a two‐stranded antiparallel *β*-sheet as reported previously [75]. (b) HT22 cells were similarly transfected with a wild-type or mutated ER*α* expression vector with single (L396R, D431Y, or Y542F) or dual (K534E/M548V) amino-acid substitutions, followed by treatment of vehicle (V), estradiol (E, 10 nM), or forskolin (F, 1 *μ*M) for 24 h. The luciferase activity (mean ± SEM) was normalized and expressed as the fold stimulation over vehicle-treated cells transfected with wild-type ER*α* (as 1 fold) from three independent experiments with triplicate samples. ^*∗*^, *P* < 0.05 vs. vehicle-treated groups (V) with the same ER expression vector.

To further dissect how the LBD mediated forskolin-induced ER*α* activation, we introduced a variety of single or double-residue mutations into the ER*α* DBD and LBD and tested their effects on the estradiol and forskolin responses (Table 1).  Figure 3 shows the sequence of ER*α* LBD with secondary structure (helixes, coil, and sheet) indicated. Among these mutations, S241E in the DBD mimics phosphorylation at this PKA phosphorylation site homologous to S236 in human ER*α*, and S523A disrupts a proposed PKA phosphorylation site homologous to S518 in human ER*α* [37, 38]. Another group of ER*α* mutants, including D356V, Y542E, Y542F, and Y542S, were selected because their equivalent residues in human or mouse ER*α* demonstrated altered transcriptional activity in response to estradiol or SERMS [39, 40]. The remaining ER*α* mutants, N353A, L408A, N460A, D478A, and H493A, were created to perturb individual helical structures of the ER*α* LBD by reducing the size of the amino-acid side chains.

Approximately 50% of the ER*α* mutants tested, including those in alpha helices (N353A, D356V, K367A, L377R, V381R, N460A, D478A, H493A, K534E, and M548V), *β* sheets (L408A), potential phosphorylation sites for PKA (S523A), or tyrosine kinases (Y531A), had similar or better transcriptional activity in response to estradiol and forskolin compared to wild-type ER*α* (Table 1, Group I). Four ER*α* mutants, S241E (mimicking potential PKA phosphorylation), E385Q, Y542E, and Y542S (around the coactivator binding sites), had no stimulation by estradiol or forskolin (Table 1, Group II). As relative to the wild-type ER*α*, loss of stimulation in E385Q, Y542E, and Y542S appeared to be due to increased basal transcriptional activity of ER*α* (2.30 ± 0.85, 5.23 ± 2.18, and 6.76 ± 2.11 folds, respectively) while S241E impaired both basal and stimulated ER*α* transcriptional activity (data not shown). Interestingly, four mutations, L396R, D431Y, Y542F, and K534E/M548V, displayed normal or enhanced stimulation by estradiol but lacked stimulation by forskolin (Figure 3(b) and Table 1, Group III). L396R, D431Y, Y542F, and K534E/M548V mutants also had lower basal transcriptional activity (0.25 ± 0.09, 0.21 ± 0.07, 0.68 ± 0.16, and 0.76 ± 0.17 folds, respectively) than wild-type ER*α* (1.00 ± 0.03 fold) (Figure 3(b)). As compared to the double mutation (K534E/M548V), ER*α* with single mutations of K534E or M548V showed partial or no suppression of forskolin-stimulated transactivation (Table 1).

### 3.4. Deletion of the ER*α* F Domain Inhibits Estradiol-Induced ER*α* Degradation, but Not Forskolin Protection from Degradation

**Figure 4:**
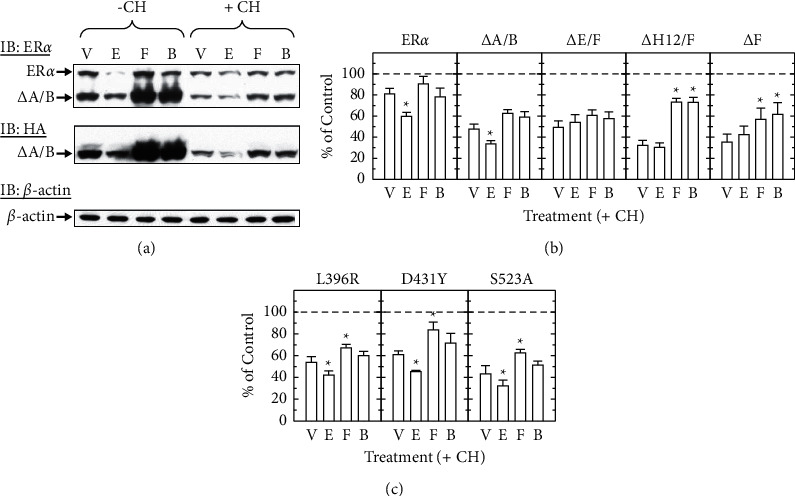
Forskolin protects both endogenous and transfected ER*α* from ligand-induced degradation in GH3 cells. (a) Cells transfected with HA-tagged ER*α* with the deletion of A/B (ΔA/B, 400 ng) were pretreated with either vehicle (-CH) or cycloheximide (+CH, 20 *μ*g/ml) for 30 min, followed by vehicle (V), estradiol (E, 10 nM), forskolin (F, 1 *μ*M), or both (B) for 6 h. Representative images show endogenous ER*α* and ΔA/B detected by immunoblotting (IB) with the ER*α* antibody (upper panel), but only ΔA/B detected specifically by the HA antibody (middle panel). Similar amounts of *β*-actin were revealed using *β*-actin antibody (lower panel). (b) Cells transfected with HA-tagged mutated ER*α* with deletion of A/B (ΔA/B), E/F (ΔE/F), F (ΔF), and helix 12/F (ΔH12/F) (400 ng) were similarly pretreated with cycloheximide, followed by vehicle (V), estradiol (E), forskolin (F), or both (B) for 6 h. Additional groups of control cells, similarly transfected with different HA-tagged ER constructs, were pretreated with vehicle (no cycloheximide) and collected in parallel, and their ER levels were used for normalization (set as 100%; dash lines). Endogenous ER*α* and HA-tagged deleted ER*α* were detected by the ER*α* and HA antibodies, respectively. (c) Cells transfected with HA-tagged, mutated ER*α* ΔA/B (L396R, D431Y, or S523A) were similarly pretreated with cycloheximide (+CH) and then received either vehicle (V), estradiol (E), forskolin (F), or both (B) and immunoblotted (IB) for HA and *β*-actin. Protein levels (mean ± SEM) of endogenous or transfected ER were normalized to the levels of *β*-actin and expressed as a percentage of the vehicle controls without cycloheximide (as 100%; dash line) from at least three experiments. ^*∗*^ depicts significantly altered ER levels over vehicle-treated controls with the same ER expression vector (V) (*P* < 0.05).

We have previously shown that endogenous ER*α* in GH3 cells is not degraded by forskolin and is even protected by forskolin against ligand-dependent degradation [8]. To identify which regions of ER*α* were required for the action of forskolin on degradation, GH3 cells were transiently transfected with HA-tagged ER*α* constructs with deletion of the A/B (ΔA/B), E/F (ΔE/F), helix 12/F (ΔH12/F), or F (Δ/F). The transfected ER*α* proteins were distinguished from endogenous ER*α* by their HA tag. As a representative blot shown in Figure 4(a), both endogenous ER*α* and transfected ΔA/B protein levels were decreased by estradiol (Lane 2) but protected from degradation by forskolin (Lanes 3 and 4). To discriminate the effects of proteolysis and translation on protein levels, cells were pretreated with cycloheximide to inhibit new protein synthesis. With the presence of cycloheximide, vehicle-treated GH3 cells showed decreased levels of endogenous ER*α* (V, 81.0 ± 5.2%) and transfected ΔA/B (V, 47.6 ± 4.7%) compared to untreated cells (100%; dash line) (Figure 4(a) and Figure 4(b), left panels), indicating that the truncated ER*α* seemed to have higher basal turnover rates than the full-length receptor. Estradiol further reduced levels of ER*α* (E, 59.7 ± 3.9%) and ΔA/B (E, 33.6 ± 3.0%) (*P* < 0.05). Forskolin not only caused no change in the levels of unliganded ERs (Groups F : ER*α* = 90.5 ± 7.2% and ΔA/B = 62.5 ± 3.5%) but also protected them from estradiol-stimulated degradation (Groups B : ER*α* = 78.3 ± 8.3% and ΔA/B = 58.9 ± 5.3%) (Figure 4(b), left panels). In turn, ER*α* mutant with a deletion of the entire LBD (ΔE/F) was not degraded by estradiol (54.0 ± 7.4%), and forskolin had no effect on levels of this truncated ER*α* protein (F, 60.6 ± 5.2% and B, 57.6 ± 6.5%) (Figure 4(b), middle panel). Deletion of the F domain (ΔF) alone or with helix 12 (ΔH12/F) resulted in a dramatic reduction in ER*α* stability in the absence of estrogen (V, 35.3 ± 7.6% and 32.3 ± 4.8% of untreated controls, respectively), rendering receptors insensitive to ligand-induced degradation (E, 42.4 ± 8.2% and 30.3 ± 4.3%), but higher levels of these truncated receptors were observed with the treatment of forskolin (F, 57.0 ± 10.5% and 73.3 ± 3.7%) or forskolin plus estradiol (B, 61.6.0 ± 11.1% and 73.0 ± 4.7%) than vehicle-treated groups (*P* < 0.05) (Figure 4(b), right panels). These data suggest that within the carboxyl-terminal region of ER*α*, the F domain is required for basal and liganded ER*α* turnover, while the E domain excluding helix 12 might be indispensable for forskolin-dependent stabilization of the receptor.

To further explore if forskolin-induced ER*α* stabilization was coupled to its transcriptional activation, we transfected GH3 cells with HA-tagged ER*α* ΔA/B carrying L396R or D431Y, the mutants that were not transcriptionally stimulated by forskolin, or S523A, the mutant that prevented ER*α* phosphorylation by PKA, and then measured the protein levels of transfected ER in response to estradiol, forskolin, or both. As compared to vehicle-treated controls, levels of ER*α* L396R (42.3 ± 3.9%), D431Y (45.3 ± 1.2%), and S523A (32.3 ± 5.2%) decreased in the presence of estradiol while forskolin alone stabilized all three ER*α* mutants (*P* < 0.05) (Figure 4(c) and S2). Forskolin inhibited ligand-dependent degradation of all three mutated ERs (L396R, 60.1 ± 3.8%; D431Y, 71.5 ± 9.1%; S523A, 51.4 ± 3.7%). Overall, forskolin protection of ER*α* from degradation seems to be independent of transcriptional activation of the receptor induced by forskolin.

### 3.5. Forskolin Protection of ER*α* Protein Does Not Result from Decreased ER*α* Ubiquitination

Ligand-bound ER*α* is ubiquitinated, resulting in the degradation of the receptor through the 26S-proteasome pathway; with inhibition of proteasome activity, ubiquitinated ER*α* can be detected as a ladder of high-molecular-weight conjugates [6, 9, 10, 25]. Therefore, we hypothesized that forskolin might prevent ER*α* ubiquitination as a means to protect it from degradation. To examine this, GH3 cells transfected with HA-tagged ubiquitin were pretreated with vehicle or MG132, a proteasome inhibitor, followed by the treatment of vehicle, estradiol, forskolin, or both for 1 or 6 h. Cells were lysed, and the extracts were subjected to immunoprecipitation with ER*α* antibody and then immunoblotted with HA antibody.

**Figure 5:**
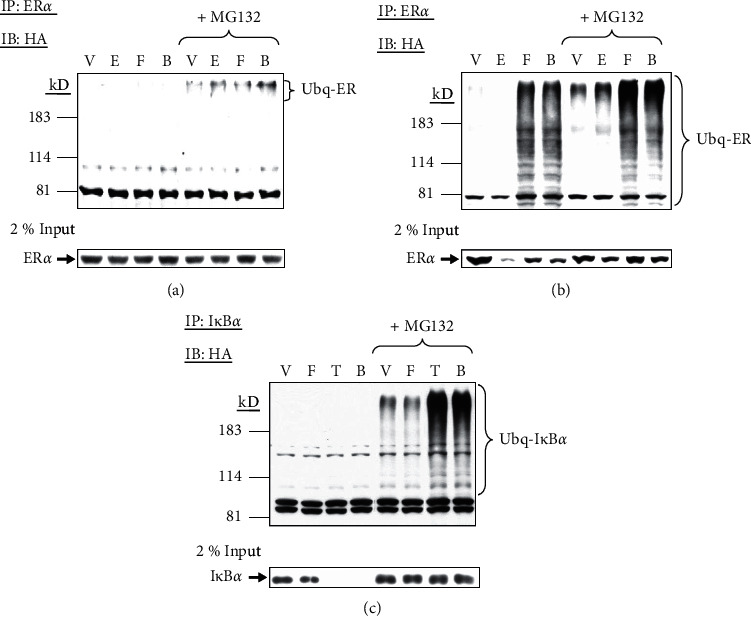
Forskolin increases ER*α*, but not I*κ*B*α*, ubiquitination. (a) GH3 cells were transfected with a HA-tagged ubiquitin expression vector (4 *μ*g). These cells were pretreated with or without MG132 (40 *μ*M) for 1 h, followed by vehicle (V), estradiol (E, 10 nM), forskolin (F, 1 *μ*M), or both (B) for 1 h. Cell lysate was immunoprecipitated (IP) with ER*α* antibody and then immunoblotted (IB) with HA to detect ubiquitinated ER*α* (Ubq-ER) (upper panel). These lysates were also subjected immunoblotting (IB) for detecting ER*α* (lower panel). (b) HA-tagged ubiquitin expressing GH3 cells were similarly pretreated with either vehicle or MG132 for 1 h followed by vehicle (V), estradiol (E), forskolin (F), or both (B) for 6 h. As described above, cell lysates were similarly immunoprecipitated (IP) for ER*α* and then immunoblotted (IB) for HA to detect ubiquitinated ER*α* (Ubq-ER) (upper panel) as well as immunoblotting (IB) for ER*α* in parallel (lower panel). (c) GH3 cells were similarly transfected with HA-tagged ubiquitin expression vector, pretreated with MG132, and then treated with vehicle (V), forskolin (F), TNF*α* (T, 100 ng/ml), or both (B) for 15 min. Cell lysate was immunoprecipitated (IP) with I*κ*B*α* antibody and then immunoblotted (IB) with HA to detect ubiquitinated I*κ*B*α* (Ubq-I*κ*B*α*) (upper panel). I*κ*B*α* was also detected by immunoblotting (IB) in these lysates (lower panel).

After one hour of treatment, low levels of ubiquitinated ER*α*, revealed by higher molecular weight bands (>183 kD), were observed in the presence of MG132 (Figure 5(a), Lanes 5–8, upper panel), and little ER*α* was degraded at the same time (Figure 5(a), lower panel). Six hours after the treatment, ER*α* in the lysate was markedly degraded in the presence of estradiol, but liganded ER*α* was protected by forskolin or MG132 (Figure 5(b), lower panel). With the presence of MG132, low, noticeable levels of polyubiquitinated ER*α* were detected in control cells, and this was enhanced by estradiol (>80 kD) (Figure 5(b), Lanes 5 and 6). Contrary to our hypothesis, forskolin greatly increased levels of ubiquitinated ER*α* in the cells in the absence or presence of estradiol (Lanes 3 and 4), and the forskolin-induced ubiquitination was seen more clearly with the pretreatment of MG132 (Lanes 7 and 8) (Figure 5(b)).

We have previously demonstrated that forskolin cannot protect TNF*α*-induced I*κ*B*α* degradation, suggesting that forskolin protection is specific to ER*α* [8]. To verify that the action of forskolin on ubiquitination was limited to ER*α* protein, we then examined the effect of forskolin on I*κ*B*α* ubiquitination. In the presence of MG132, a basal level of ubiquitinated I*κ*B*α* was detected in vehicle-treated cells (Figure 5(c), Lane 5). As I*κ*B*α* was protected from TNF*α*-induced degradation by MG132, a robust increase in the accumulation of ubiquitinated I*κ*B*α* was observed (Figure 5(c), Lane 7). Forskolin did not alter the basal or TNF*α*-induced I*κ*B*α* ubiquitination in GH3 cells (Figure 5(c), Lanes 6 and 8), confirming that forskolin-stimulated ubiquitination was ER*α*-specific, not a global effect.

### 3.6. ER*α* Ubiquitination Is Reduced by the K48R Ubiquitin Mutation after All Treatments

**Figure 6:**
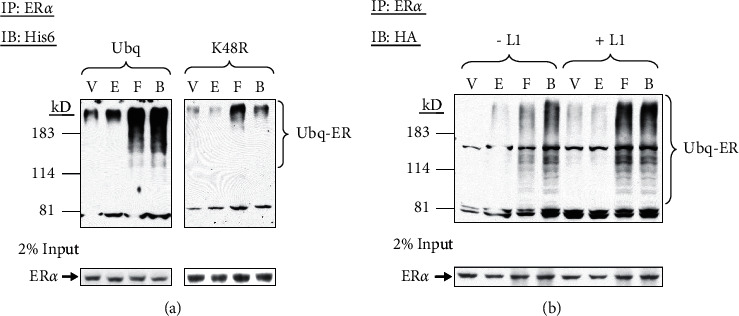
The K48R mutation in ubiquitin reduces estradiol and forskolin-induced ER*α* ubiquitination. (a) GH3 cells were transfected with His6-tagged, wild-type (Ubq), or K48R mutated (K48R) ubiquitin expression vector (4 *μ*g). Cells were pretreated with MG132 (40 *μ*M) for 1 h followed by vehicle (V), estradiol (E, 10 nM), forskolin (F, 1 *μ*M), or both (B) for 6 h. Cell lysates were subjected to immunoprecipitation (IP) with ER*α* antibody, and analyzed by immunoblotting (IB) with antibody against His6 to detect ubiquitinated ER*α* (Ubq-ER) (upper panel). ER*α* was also detected by immunoblotting (IB) with the ER*α* antibody from these lysates (lower panel). (b) GH3 cells transfected with HA-tagged ubiquitin expression vector (4 *μ*g) were pretreated with vehicle (-L1) or the UCH-L1 inhibitor (+L1, 1 *μ*M) for 1 h followed by vehicle (V), estradiol (E), forskolin (F), or both (B) for 6 h. Ubiquitinated ER*α* (Ubq-ER) was detected from cell lysates with immunoprecipitated (IP) for ER*α* and then immunoblotted (IB) for HA (upper panel) while ER*α* was also detected by immunoblotting (IB) of ER*α* in parallel (lower panel).

Ubiquitination on ubiquitin K48 is essential for the polyubiquitin chain assembly with at least four ubiquitin monomers to mark the substrate proteins for degradation via the proteasome pathway while ubiquitination through other lysine residues of ubiquitin (K11, K29, and K63) alters other biological activities such as protein sorting, translation, and DNA repair [50]. To examine whether forskolin-induced ER*α* ubiquitination was mediated exclusively by K48, we transfected GH3 cells with vectors containing His6-tagged, wild-type, or K48R ubiquitin; K48R mutant should prevent conjugation of another ubiquitin at the preferred K48 position but not interfere the polyubiquitin chains via other lysines [44]. In the presence of wild-type ubiquitin, treatment with vehicle or estradiol slightly increased ER*α* ubiquitination, and treatment with forskolin increased both unliganded and liganded ER*α* ubiquitination substantially (Figure 6(a), left panel). Overexpression of K48R ubiquitin abolished vehicle- and estradiol-induced ER*α* ubiquitination, and greatly reduced levels of forskolin-stimulated ubiquitinated ER*α* (Figure 6(a), right panel). This finding confirms that the formation of K48-linked ubiquitin chains is required for estradiol- and forskolin-mediated ER*α* ubiquitination.

Besides ubiquitin ligases that add ubiquitin to substrate proteins, deubiquitinases negatively regulate ubiquitination by “trimming” or removing polyubiquitin chains [51]. UCH-L1 is abundant in the mouse pituitary (exclusively in gonadotrophs and lactotrophs) and brain [52]. Thus, we treated GH3 cells, transfected with HA-tagged ubiquitin expression vector, with the inhibitor of UCH-L1 to determine whether this deubiquitinase might contribute to estradiol- and/or forskolin-induced ER*α* ubiquitination. We found that this treatment slightly enhanced forskolin-induced ubiquitination of unliganded and liganded ER*α* (Figure 6(b), Lanes 7 and 8). Our result indicates that deubiquitinating enzymes, or at least UCH-L1, may not play a direct role in regulating ER*α* ubiquitination induced by estradiol or forskolin.

### 3.7. Forskolin-Stimulated ER*α* Ubiquitination Requires the Carboxyl-Terminal F Domain

**Figure 7:**
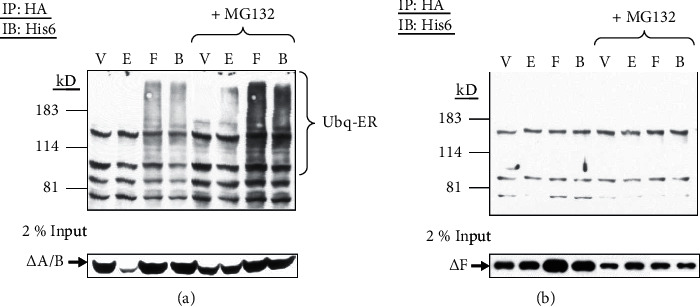
The F region is required for forskolin-stimulated ER*α* ubiquitination. (a) GH3 cells were transfected with His6-tagged ubiquitin and HA-tagged ER*α*ΔA/B expression vectors (2 *μ*g each). Cells were pretreated with MG132 (40 *μ*M) for 1 h followed by vehicle (V), estradiol (E, 10 nM), forskolin (F, 1 *μ*M), or both (B) for 6 h. Cell lysates were subjected to immunoprecipitation (IP) with HA and analyzed by immunoblotting (IB) with the His6 antibody to detect ubiquitinated ER*α* ΔA/B (Ubq-ER). In parallel, the same lysates (10 *μ*l each) were also immunoblotted (IB) with the HA antibody (lower panel). (b) GH3 cells transfected with His6-tagged ubiquitin and HA-tagged deleted ER*α* (ΔF) expression vectors (2 *μ*g each) were similarly pretreated with MG132, followed by vehicle (V), estradiol (E), forskolin (F), or both (B) for 6 h. Cell lysates were similarly immunoprecipitated (IP) for HA and then immunoblotted (IB) for His6 to detect ubiquitinated ER*α*ΔF (upper panel) as well as immunoblotted (IB) with HA antibody for ER*α*ΔF in parallel (lower panel).

We next used rat ER*α* deletion constructs to assess which specific region was required for estradiol- or forskolin-stimulated ubiquitination. GH3 cells were transfected with HA-tagged ER*α* deletion construct (ΔA/B or ΔF) and His6-ubiquitin, then similarly pretreated with MG132, followed by the treatments of vehicle, estradiol, forskolin, or both. Using immunoprecipitation with HA and immunoblotting with His6, we demonstrated that with deletion of the A/B domain (ΔA/B), the pattern of ubiquitinated amino-terminally truncated ER*α* was similar to that of the full-length receptor (Figure 7(a)), indicating that the A/B domain does not regulate the ubiquitination step of ER*α* or serve as the ubiquitination site. In contrast, deletion of the F domain resulted in less detectable ubiquitinated ER*α* with any treatment (Figure 7(b)). Our data show that the F domain of ER*α* is required for its ubiquitination elicited by estradiol, forskolin, or both.

### 3.8. Forskolin Protects ER*β* from Estradiol-Dependent Degradation in the Absence of Transcriptional Activation

**Figure 8:**
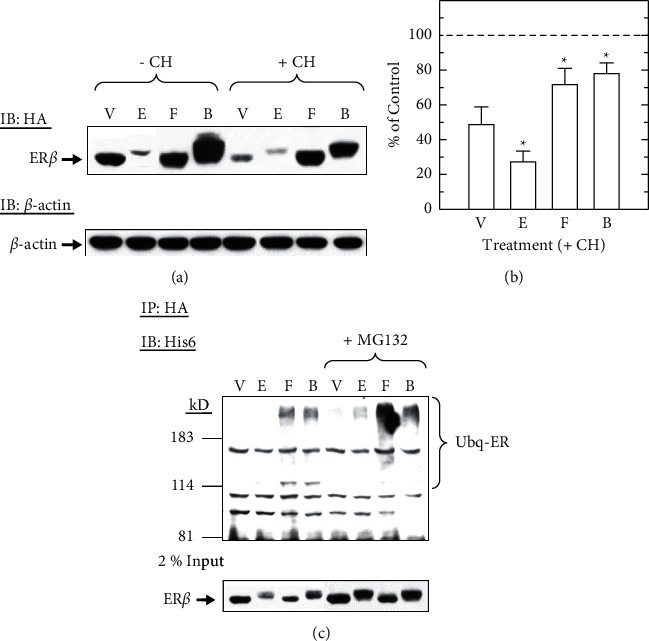
Forskolin protects transfected ER*β* from ligand-induced degradation and increases ER*β* ubiquitination. (a) GH3 cells transfected with HA-tagged ER*β* (400 ng) were pretreated with either vehicle (-CH) or cycloheximide (+CH, 20 *μ*g/ml) for 30 min, followed by vehicle (V), estradiol (E, 10 nM), forskolin (F, 1 *μ*M), or both (B) for 6 h. ER*β* and *β*-actin were both detected by immunoblotting (IB) with the antibodies against HA (upper panel) and *β*-actin (lower panel), respectively. (b) GH3 cells transfected with HA-tagged, ER*β* were similarly pretreated with cycloheximide and then received either vehicle (V), estradiol (E), forskolin (F), or both (B) and immunoblotted (IB) for HA and *β*-actin. Relative ER*β* protein levels (mean ± SEM) were normalized to the levels of *β*-actin and expressed as a percentage of the untreated control (as 100%, dash line). (c) GH3 cells were transfected with HA-tagged ER*β* and His6-tagged ubiquitin (2 *μ*g each) and then pretreated with vehicle or MG132 (40 *μ*M) for 1 h followed by vehicle (V), estradiol (E), forskolin (F), or both (B) for 6 h. Cell lysates were immunoprecipitated (IP) with HA antibody and then immunoblotted (IB) for His6 to detect ubiquitinated ER*β* (Ubq-ER). In parallel, the same lysates (10 *μ*l each) were immunoblotted (IB) with the HA antibody (lower panel). ^*∗*^ depicts significantly altered ER levels or transcriptional activity as compared to vehicle-treated controls (V) (*P* < 0.05).

Although ER*β* was not transactivated by forskolin (Figure 1(b)), with a high homology to ER*α* in the LBD, we tested if forskolin protected ER*β* from basal and ligand-induced degradation. We found that in the pretreatment of cycloheximide, unliganded ER*β* protein levels were decreased to 48.7 ± 10.0% of control levels, and in response to estradiol, a further reduction (27.3 ± 6.1%) was observed (Figures 8(a) and 8(b)). Unliganded and liganded ER*β* were similarly protected from degradation by forskolin (F, 71.1 ± 9.3% and B, 78.0 ± 6.3%, respectively) (Figures 8(a) and 8(b)). In addition, estradiol-bound ER*β* appeared to be slightly upshifted, indicating that posttranslational modifications might occur on ER*β* after estradiol binding (Figure 8(a), Lanes 2, 4, 6, and 8). This observation suggests that forskolin protection of ERs from degradation seems to be independent of transcriptional activation of the receptor induced by forskolin.

To examine whether forskolin protection of ER*β* from degradation was associated with increased ubiquitination, GH3 cells were transfected with HA-ER*β* and His6-ubiquitin expression vectors, then pretreated with either vehicle or MG132, followed by vehicle, estradiol, forskolin, or both. In the absence of MG132, estradiol rapidly resulted in ER*β* degradation, which made it difficult to detect ubiquitinated ER, whereas ubiquitination of unliganded and liganded ER*β* was observed after forskolin treatment alone and with estradiol, respectively (Figure 8(c), Lanes 2–4). After the proteolytic activity of the 26S proteasome was inhibited by MG132, ubiquitinated ER*β* in estradiol-treated cells became obvious and was greatly increased by forskolin regardless of estradiol (Figure 8(c), Lanes 6–8). Thus, similar to ER*α*, the ability of forskolin to protect ER*β* from degradation does not occur by decreasing ER ubiquitination or depend on stimulation of ER-mediated transcription.

## 4. Discussion

Our previous study has shown that in ER*α*-positive, rat GH3 pituitary cells, estradiol- and forskolin-stimulated ER transcriptional activations differ in the time courses of transcriptional activation, coupling with receptor turnover, and responses to proteasome inhibition [8], suggesting distinct mechanisms by which estradiol and forskolin elicit ER*α*-mediated transcription. ER*α* transcriptional activity is mediated by the amino-terminal AF-1 (A/B domain) and the carboxyl-terminal AF-2 (E/F domain) regions [20, 53, 54], and our deletion mutation experiments demonstrate that similar to estradiol, forskolin stimulation of rat ER*α* transcriptional activity occurs mainly through the E region, particularly the helix 12 (Figure 2(b)). With the requirement of AF-2, estrogen- and forskolin-dependent activations of ER*α* are likely to share some common mechanisms. In support of this, overexpression of the p160 coactivators, including steroid receptor coactivator-1 (SRC-1), transcription intermediary factor-2 (TIF2), and receptor associated coactivator-3 (RAC3), as well as the general coactivators, p300 and CREB-binding protein (CBP), and coactivator-associated arginine methyltransferase 1 (CARM1) enhances ER*α*-dependent transcription activated by estradiol and cAMP [55, 56]. These findings suggest that estrogen-bound and forskolin-activated ER*α* may undergo a similar conformational change that forms a binding surface to recruit and interact with coactivators as well as other factors in the basal transcriptional machinery through the helix 12.

Despite the requirement of AF-2 for the responses to estradiol and forskolin, ER*α* transcriptional activity stimulated by forskolin and estrogen can be differentiated through the mutations of L396R, D431Y, Y542F, or K534E/M548V on ER*α* LBD (Figure 3(b) and Table 1). Two other mutations, G400V and S464A, were reported earlier to make the human ER*α* unresponsive to cAMP/PKA while the ligand-dependent transactivation remained intact [55, 57]. These residues are scattered over several helices, including helices 6 (L396), 8 (D431), 10 (S469 in rat; S464 in human), 11 (K534), and 12 (Y542 and M548) as well as the *β*-sheet (G405 in rat; G400 in human) of the ER*α* LBD (Figure 3(a)). While normal interactions between ER*α* and cofactors are suggested to be responsible for forskolin-induced activation of ER*α*, comparing to the crystal structure of human ER*α* LBD complexed with estradiol [58], none of these residues, except M548, constitute the hydrophobic groove on the interacting surface of the ER*α* LBD for coactivator binding. Therefore, we speculate that along with G405 and S469, the two corresponding residues of human ER*α* G400 and S464, L396, D431, K534, Y542, and M548 might define a novel regulatory surface specific for the interaction of unliganded rat ER*α* with coregulatory proteins after forskolin stimulation, resulting in distinct transcriptional programs for the ligand-dependent and independent ER activation.

Regarding the molecular mechanism downstream of forskolin stimulation, we have previously shown that forskolin increases ER transcriptional activity exclusively through the cAMP-PKA pathway in ER*α*-positive pituitary cells [7, 8]. Direct phosphorylation of ER*α* was originally proposed to mediate the ligand-independent activation by forskolin because PKA phosphorylates human ER*α* at S236 and S305 [38, 59, 60]. Phosphorylation of S236, located within the DBD, inhibits dimerization and DNA binding of unliganded ER*α*, and a glutamic acid (S236E), not alanine (S236A), substitution impairs ER*α* dimerization, which abolishes the estradiol- and PKA-stimulated transactivation [38]. The inhibitory effect of this mutation on ER*α* transcriptional activity is confirmed at the rat equivalent (S241E) (Table 1). A previous study showed that ligand-independent ER*α* activation was stimulated by low and intermediate levels of transfected PKA catalytic subunits, but suppressed by high levels, suggesting that the phosphorylation of ER*α* S236 and its inhibitory effects may occur only at higher levels of cAMP/activated PKA [55]. S305 of human ER*α*, located in the hinge region, is also phosphorylated by PKA, but an alanine substitution of S305 (S305A) does not abolish ER*α*-mediated transcription elicited by cAMP or forskolin/IBMX [55, 61]. Apart from S236 and S305, S518 in the LBD is a potential PKA phosphorylation site because of being embedded in a PKA recognition motif. Mutation of the equivalent residue in rat ER*α* (S523A) had no effect on the estradiol- or forskolin-elicited ER*α* transcriptional activity (Table 1). On the other hand, L396, D431, K534, Y542, and M548, responsible for the forskolin-stimulated ER*α* activation, are not phosphorylated residues for PKA or near a consensus substrate motif of PKA. Instead, Carascossa et al. reported that PKA phosphorylated CARM1, which allows the direct binding of CARM1 to unliganded ER*α* LBD to mediate cAMP activation of ER*α* [55]. While direct phosphorylation of rat ER*α* cannot be completely excluded, PKA-dependent phosphorylation of ER-interacting coregulatory proteins, enhancing their recruitment to the receptor, appears to be the molecular basis for the ligand-independent ER*α* activation by forskolin.

In the present study, we have also observed that the forskolin-stimulated transactivation is specific to rat ER*α*, but not ER*β* (Figure 1(a)), consistent with two previous studies on human ER*α* and ER*β* [55, 56]. Since AF-2 has the importance for ER*α* activation in response to forskolin, distinct amino‐acid compositions at this region might render the ERs with subtype‐specific properties in conveying forskolin signaling. However, the five residues (L396, D431, K534. Y542, and M548) that are required for forskolin stimulation of rat ER*α* transactivation are all conserved in ER*β* (L362, D397, K499, Y507, and M513), suggesting that these amino acids are unlikely to be the molecular determinants of ER subtype-specific activation by forskolin. On the other hand, human ER*β* contains an alanine (A) in the position corresponding to S464 in ER*α*, and alanine substitution (S464A) abolishes the transcriptional response of ER*α* to cAMP and the interaction of ER*α* with CARM1 [55]. The serine and alanine residues in the two ER subtypes are conserved from human to rat (S469 in ER*α* and A435 in ER*β*, respectively), so they might be responsible for ER*α*-specific activation by forskolin. Besides the LBD, the F domains of the rat ER*α* and ER*β* are different in both length (42 versus 28 amino acids) and sequence identity (17%). Replacing the F domain of human ER*α* with that from ER*β* eliminates estradiol-induced transcriptional activity of the receptor at an AP-1 site [62]. Since deletion of the F domain does not abolish forskolin stimulation of rat ER*α* (Figure 2(b)), the F domain of ER*β* might inhibit the receptor from being transcriptionally activated by forskolin, which needs to be elucidated by the removal and replacement of the ER*β* F domain.

Besides ERs and their coactivators, the promoter complexity of the reporter gene may also play an important role in defining differential ligand-independent activation between ER*α* and ER*β* [27, 56, 63]. A prior report showed that forskolin/IBMX stimulated the transcriptional activities of both human ER*α* and ER*β* on a complex promoter that contained a 12-O-tetradecanoylphorbol-13-acetate response element (TRE) located upstream to an ERE, and mutation of the TRE abolished the ligand-independent activation of ER*β*, but not ER*α* [56]. This finding may help explain why forskolin stimulates transcriptional activity of rat ER*α*, but not ER*β* (Figure 1(a)), as we use a simple model reporter with the promoter containing only two EREs upstream of the rat prolactin TATA box to evaluate ER-mediated transcription. In addition, we have also observed that the same forskolin treatment fails to stimulate transfected rat ER*α* in ER-negative COS cells derived from monkey kidney tissue (not shown). Together, these results suggest that differential forskolin stimulation of ER transcriptional activity between ER*α* and ER*β* might be determined by ER subtypes, promoter contents, and cell contexts.

Estradiol-stimulated ER*α* transactivation is coupled to increased turnover of the receptor through the ubiquitin-proteasome system [6, 9, 20]. The current study confirms that ligand binding increases ubiquitination and degradation of rat ER*α* and further demonstrates that both molecular events require the F region (Figures 4(b) and 7(b)). On examining the sequence of the F domain of rat ER*α*, we discover a PEST-like sequence (amino acid 560–582: RMGVPPEEPSQSQLTTTSSTSAH) with a high score of 9.23 as predicted by ePESTfind (http://emboss.bioinformatics.nl/cgi-bin/emboss/epestfind). Many short-lived proteins degraded by the ubiquitin-proteasome pathway contain a PEST motif, enriched in proline, glutamate, serine, and threonine, and the PEST sequence serves as a phosphodegron to recruit ubiquitin E3 ligases for protein ubiquitination and degradation. The PEST domain has been shown to mediate I*κ*B*α* ubiquitination and degradation induced by TNF*α* [64, 65]. Deletion of the F domain had no effect on ligand-induced human ER*α* degradation [6, 66]. As compared to the rat, the F domain of human ER*α* displays only 60% homology and lacks the putative PEST motif, which might be responsible for the discrepancy in the role of the F domain in the regulation of liganded ER*α* stability between rat and human.

Unlike estradiol, forskolin not only stimulates ER*α* transcriptional activation without being accompanied by protein degradation but also enhances estradiol-dependent ER*α* activation with the protection of liganded receptor from proteolysis [8]. Several findings from the current study further support our previous observation, demonstrating that the effect of forskolin on ER*α* protein stability can be uncoupled from that on transactivation. First, forskolin protects both ER*α* and ER*β* from estradiol-induced degradation, even though only the former is transcriptionally activated by forskolin (Figures 1(b), 4, and 8). Second, two ER*α* LBD mutants, L396R and D431Y, lacking forskolin-stimulated transcriptional activity, are protected by forskolin from both basal turnover and estradiol-induced degradation (Figures 3(b) and 4(c)). Third, deletion of helix 12 and the F domain disrupts estradiol- and forskolin-induced ER*α* transactivation while it has no effect on forskolin protection of the receptor from proteolysis (Figures 2(b) and 4(b)). Thus, these observations suggest that forskolin stimulation of ER transcriptional activity and its protection of ER from degradation are possibly mediated by distinct mechanisms.

The ligand-bound ER*α* and ER*β* both are degraded through the ubiquitin-proteasome pathway, so forskolin protection of ERs from degradation prompts us to examine the degree of receptor ubiquitination after estradiol and forskolin treatment. In contrast to our original hypothesis, forskolin drastically increases ubiquitination of ER*α* and ER*β* regardless of the presence or absence of estrogen, which appears to be more robust than that induced by estradiol alone (Figures 5(b) and 8(c)). Forskolin action on ER ubiquitination is protein-specific because the same treatment does not alter TNF*α*-induced ubiquitination of I*κ*B*α* (Figure 5(c)). In addition, the F domain is found to mediate both estradiol- and forskolin-stimulated ER*α* ubiquitination while deletion of the A/B domain does not alter the ubiquitination status of liganded or unliganded ER*α* (Figure 7). Ubiquitination takes place on the lysine residues of the target proteins [18]. Rat ER*α* contains 28 lysine residues, but none of those are located within the F domain. Thus, instead of being the substrate site for ubiquitin conjugation, the F region may provide an interaction site that recruits ubiquitin ligases and other components of the ubiquitination machinery. Future investigation will be needed to determine which of the ER*α* lysines are ubiquitinated in response to estradiol and/or forskolin and to identify the specific ubiquitin ligases involved in these processes.

The signal for the substrate proteins targeted for the proteasomal degradation has been characterized as a polyubiquitination chain that consists at least four ubiquitin monomers conjugated through K48 of ubiquitin, whereas monoubiquitination as well as polyubiquitination through other lysine residues (K11, K29, and K63) within the ubiquitin molecule may involve other cellular functions, such as protein sorting and DNA repair [13, 15–18]. In the current study, the size of ubiquitinated ER*α* (>114 kD) and requirement of K48 suggest that estradiol and forskolin both stimulate ER*α* polyubiquitination through K48-linked ubiquitin chains (Figure 6(a)). This agrees with the observation by Iizuka et al., showing that the overexpression of K48R mutant markedly decreased human ER*α* polyubiquitination [67]. Similar to ER*α*, the polyubiquitin chains of human ER*β* are also linked via K48 [68]. Besides the addition of ubiquitin, deubiquitinating enzymes, including UCH and ubiquitin-specific processing protease (USP), also play important roles in regulation of ubiquitination by removing the ubiquitin from the substrate proteins or the ubiquitin chains [51, 69]. Since inhibition of UCH-L1 slightly enhances ubiquitination of unliganded and liganded ER*α*, but cannot mimic the action of forskolin (Figure 6(b)), we conclude that UCH-regulated deubiquitination alone may play a small role in forskolin stimulation of ER*α* ubiquitination. Moreover, the degrees and patterns of ER ubiquitination are different after estradiol and forskolin treatments although both treatments increase polyubiquitination. Increased polyubiquitination at the same sites and/or mono- or polyubiquitination on additional sites within the ER protein may account for such differences.

It has been reported that along with ubiquitin ligases, 19S regulatory components of the proteasome are recruited with ER*α* on the pS2 promoter, which might lead to the coupling of estrogen-regulated ER*α* proteolysis and transcription [19, 20]. Our current work showed that forskolin stimulation of ER*α* transcriptional activity did not require proteasome activity (Figure 1(c)). Thus, we speculate that uncoupling of forskolin-stimulated ER ubiquitination from degradation might be caused by failure of recruitment of proteasome components. Besides the 26S proteasome, a previous study reported that the lysosome-dependent degradation pathway also contributed to the estradiol-dependent ER*α* breakdown in MCF-7 cells [70]. Totta and colleagues observed that cytoplasmic ER*α* was routed to lysosomes and then endosomes in an estradiol-dependent manner, and inhibition of lysosomal function increased liganded ER*α* accumulation. Interestingly, the lysosome-mediated degradation is not required for ER*α*-regulated, ERE-containing gene transcription. Thus, forskolin protection of liganded and unliganded ERs from degradation might in part take place in the lysosomes or by preventing ER from being routed to lysosomes.

Similar to transcriptional activity, forskolin protection of ER*α* from degradation is also mediated through the PKA pathway [8]. Rolli-Derkinderen et al. demonstrated that phosphorylation of RhoA at S188 protected RhoA from ubiquitin-mediated proteasomal degradation [71]. Mutation of the potential PKA phosphorylation site (S523A) in the rat ER*α* LBD has no effect on receptor turnover (Figure 4(c)), but other PKA phosphorylation sites located in the LBD might mediate the protective effect of forskolin on ER*α* degradation. Meanwhile, instead of ER*α* itself, ER-interacting proteins might also be the targets for PKA to increase the stability of the receptor. As mentioned above, several steroid receptor coactivators can be phosphorylated in response to cAMP [55, 72, 73]. Two previous studies have shown that PKA phosphorylates the CARM1 and lysine-specific histone demethylase 1 (LSD1), which induces their recruitment to the unliganded ER*α* [55, 73]. In addition, PKA-stimulated ubiquitination has been observed in GRIP-1, a steroid receptor coactivator although this was associated with increased protein degradation [74]. Thus, forskolin action on ER*α* transcriptional activity, protection, and ubiquitination might be contributed by PKA-mediated phosphorylation of ER-interacting proteins rather than ER*α* itself.

Our studies have characterized several unique features and novel mechanisms for ligand-independent activation and/or protection of ERs by forskolin. Overall, because forskolin-stimulated pathways stabilize both ER subtypes, and because the amount of ER directly correlates to the transcriptional and biological response [46, 75], these signaling pathways will directly impact the ability of specific cells and tissues to respond to ligands as well as to ligand-independent pathways physiologically and pathologically.

## Figures and Tables

**Table 1:** Transcriptional activity of ER*α* and its mutants in response to estradiol or forskolin^a^.

Residue	Helix^b^	Treatment		
		Vehicle	Estradiol	Forskolin
Wild-type ER*α*		1.00 ± 0.03	3.60 ± 0.31^*∗*^	4.19 ± 0.35^*∗*^

Group I : No change/increase of estradiol or forskolin stimulation:				
N353A	3	1.00 ± 0.07	6.76 ± 1.94^*∗*^	9.69 ± 2.40^*∗*^
D356V	3	1.00 ± 0.09	9.51 ± 0.58^*∗*^	11.32 ± 0.47^*∗*^
K367A	3	1.00 ± 0.02	2.31 ± 0.05^*∗*^	3.07 ± 0.36^*∗*^
L377R	5	1.00 ± 0.03	8.17 ± 2.61^*∗*^	6.32 ± 0.67^*∗*^
V381R	5	1.00 ± 0.02	2.79 ± 0.71^*∗*^	2.27 ± 0.16^*∗*^
L408A	S1/S2	1.00 ± 0.02	3.96 ± 0.56^*∗*^	5.25 ± 1.11^*∗*^
N460A	9	1.00 ± 0.04	7.01 ± 2.07^*∗*^	12.16 ± 4.26^*∗*^
D478A	10	1.00 ± 0.04	6.43 ± 1.82^*∗*^	9.43 ± 3.01^*∗*^
H493A	10	1.00 ± 0.05	8.93 ± 0.61^*∗*^	12.94 ± 1.41^*∗*^
S523A	11	1.00 ± 0.06	3.01 ± 0.64^*∗*^	4.16 ± 0.64^*∗*^
Y531A	11	1.00 ± 0.03	11.10 ± 1.90^*∗*^	15.10 ± 5.01^*∗*^
K534E	11	1.00 ± 0.05	13.98 ± 1.51^*∗*^	5.61 ± 0.54^*∗*^^#^
M548V	12	1.00 ± 0.12	13.83 ± 1.82^*∗*^	11.45 ± 2.00^*∗*^

Group II : Loss of both estradiol and forskolin stimulation:				
S241E	DBD	1.00 ± 0.10	1.79 ± 0.41	1.24 ± 0.12
E385Q	5	1.00 ± 0.06	1.60 ± 0.21	1.96 ± 0.37
Y542E	12	1.00 ± 0.17	1.54 ± 0.39	1.30 ± 0.34
Y542S	12	1.00 ± 0.14	0.98 ± 0.09	0.66 ± 0.10

Group III : Loss of forskolin stimulation:				
L396R	6	1.00 ± 0.06	17.79 ± 1.50^*∗*^	0.98 ± 0.09^#^
D431Y	8	1.00 ± 0.03	20.5 ± 6.23^*∗*^	1.56 ± 0.20^#^
K534E/M548V	11/12	1.00 ± 0.06	7.70 ± 1.11^*∗*^	1.20 ± 0.10^#^
Y542F	12	1.00 ± 0.11	3.90 ± 1.00^*∗*^	1.61 ± 0.21^#^

^a^HT22 cells were transfected with pGL3-ERE2-luciferase and individual wild-type or ER*α* mutants as described in Methods and treated for 24 h with either vehicle, 10 nM estradiol, or 1 *μ*M forskolin. At least three separate experiments with triplicate samples per group were performed. Normalized luciferase activities were calculated as relative to the vehicle-treated controls of individual ER vectors (as 1 fold) and expressed as mean ± SE. ^b^The numbers indicate which helices the mutated residues reside as corresponding to the human ER*α* ligand-binding domain (LBD) reported by Pavlin et al. [49]. DBD, DNA binding domain; S1/S2, two‐stranded antiparallel *β*-sheet. ^*∗*^and ^#^ denote significant differences (*P* < 0.05) as compared to vehicle- and estradiol-treated groups, respectively, using one-way ANOVA and Tukey's wholly significant difference (WSD) post hoc test.

## Data Availability

The data that support the findings of this study are available on request from the corresponding author, Houng-Wei Tsai (houng-wei.tsai@csulb.edu).

## Supplementary Materials

Supplementary Materials
*Figure S1.* Representative immunoblots showing the expression of transfected wild-type (WT) and mutated ER*α* in ER-negative mouse hippocampal HT22 cells. Cells were transfected with an expression vector of WT or mutated ER*α* (400 ng) for 24 h. Cells were then lysed, and approximately 30 *μ*g of protein lysates was separated on polyacrylamide-SDS gels, transferred to nitrocellulose membranes, and subjected to immunoblotting with ER*α* antibody. C, controlled cells transfected with an empty expression vector. *Figure S2.* Representative immunoblots showing the effects of estradiol, forskolin, or both on protein levels of mutated ER*α*, including ΔE/F, ΔH12/F, ΔF, and ΔA/B (L396R, D431Y, or S523A), in GH3 cells. Cells, transfected with HA-tagged mutated ER*α* (400 ng), were pretreated with either vehicle (C) or cycloheximide (+CH, 20 *μ*g/ml) for 30 min. The cycloheximide-pretreated cells were then treated with vehicle (V), estradiol (E, 10 nM), forskolin (F, 1 *μ*M), or both (B) for 6 h. Transfected ER*α* and endogenous *β*-actin were detected by immunoblotting with the antibodies against HA and *β*-actin, respectively.
