# Supplementary figures and images for: Forskolin Stimulates Estrogen Receptor (ER) α Transcriptional Activity and Protects ER from Degradation by Distinct Mechanisms
Source: Int J Endocrinol. 2022 May 9;2022:7690166. doi: 10.1155/2022/7690166 (PMC9110234; doi:10.1155/2022/7690166)

Supplement 1.

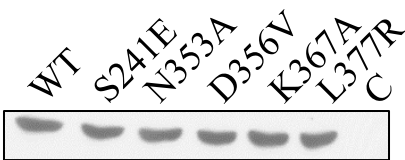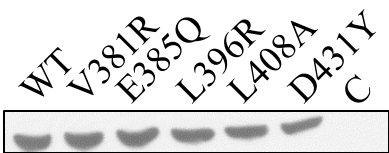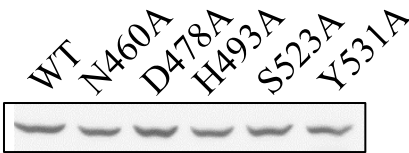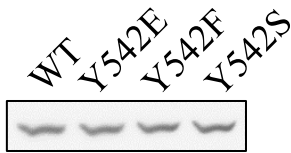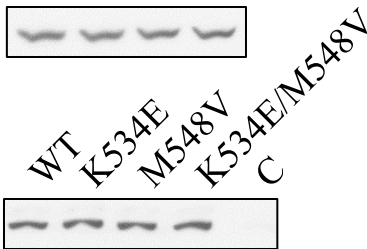

Supplement: Supplementary Materials — Figure S1. Representative immunoblots showing the expression of transfected wild-type (WT) and mutated ERα in ER-negative mouse hippocampal HT22 cells. Cells were transfected with an expression vector of WT or mutated ERα (400 ng) for 24 h. Cells were then lysed, and approximately 30 μg of protein lysates was separated on polyacrylamide-SDS gels, transferred to nitrocellulose membranes, and subjected to immunoblotting with ERα antibody. C, controlled cells transfected with an empty expression vector. Figure S2. Representative immunoblots showing the effects of estradiol, forskolin, or both on protein levels of mutated ERα, including ΔE/F, ΔH12/F, ΔF, and ΔA/B (L396R, D431Y, or S523A), in GH3 cells. Cells, transfected with HA-tagged mutated ERα (400 ng), were pretreated with either vehicle (C) or cycloheximide (+CH, 20 μg/ml) for 30 min. The cycloheximide-pretreated cells were then treated with vehicle (V), estradiol (E, 10 nM), forskolin (F, 1 μM), or both (B) for 6 h. Transfected ERα and endogenous β-actin were detected by immunoblotting with the antibodies against HA and β-actin, respectively. [file 7690166.f1.zip › 7690166.f1/7690166-Tsai_S1.pdf]

Supplement 2.

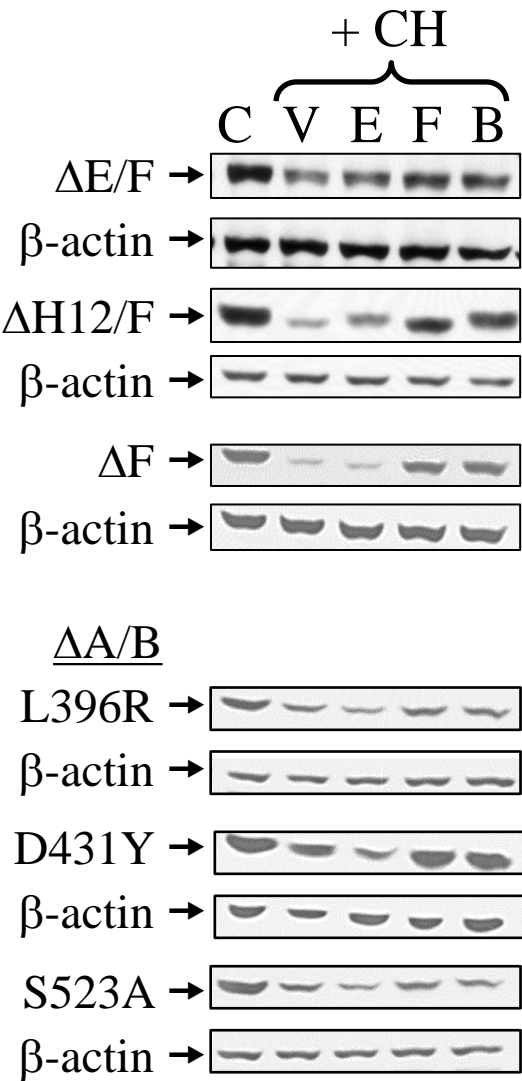

Supplement: Supplementary Materials — Figure S1. Representative immunoblots showing the expression of transfected wild-type (WT) and mutated ERα in ER-negative mouse hippocampal HT22 cells. Cells were transfected with an expression vector of WT or mutated ERα (400 ng) for 24 h. Cells were then lysed, and approximately 30 μg of protein lysates was separated on polyacrylamide-SDS gels, transferred to nitrocellulose membranes, and subjected to immunoblotting with ERα antibody. C, controlled cells transfected with an empty expression vector. Figure S2. Representative immunoblots showing the effects of estradiol, forskolin, or both on protein levels of mutated ERα, including ΔE/F, ΔH12/F, ΔF, and ΔA/B (L396R, D431Y, or S523A), in GH3 cells. Cells, transfected with HA-tagged mutated ERα (400 ng), were pretreated with either vehicle (C) or cycloheximide (+CH, 20 μg/ml) for 30 min. The cycloheximide-pretreated cells were then treated with vehicle (V), estradiol (E, 10 nM), forskolin (F, 1 μM), or both (B) for 6 h. Transfected ERα and endogenous β-actin were detected by immunoblotting with the antibodies against HA and β-actin, respectively. [file 7690166.f1.zip › 7690166.f1/7690166-Tsai_S2.pdf]
